# Multifunctional fish gelatin hydrogel inverse opal films for wound healing

**DOI:** 10.1186/s12951-022-01564-w

**Published:** 2022-08-02

**Authors:** Xinyue Cao, Zhuohao Zhang, Lingyu Sun, Zhiqiang Luo, Yuanjin Zhao

**Affiliations:** 1 Department of Rheumatology and Immunology, Nanjing Drum Tower Hospital, School of Biological Science and Medical Engineering, Southeast University, Nanjing, 210096 China; 2 Oujiang Laboratory (Zhejiang Lab for Regenerative Medicine, Vision and Brain Health), Wenzhou Institute, University of Chinese Academy of Sciences, Wenzhou, 325001 Zhejiang China

**Keywords:** Fish gelatin, Structural color, Sensor, Wound healing, Patch, Hydrogel

## Abstract

**Background:**

Wound healing has become a worldwide healthcare issue. Attempts in the area focus on developing patches with the capabilities of avoiding wound infection, promoting tissue remolding, and reporting treatment status that are of great value for wound treatment.

**Results:**

In this paper, we present a novel inverse opal film (IOF) patch based on a photo-crosslinking fish gelatin hydrogel with the desired features for wound healing and dynamic monitoring. The film with vibrant structure colors was constructed by using the mixture of fish gelatin methacryloyl, chitosan, and polyacrylic acid (PAA) to replicate colloidal crystal templates. As the structures of these natural biomolecules are well-retained during the fabrication, the resultant IOF was with brilliant biocompatibility, low immunogenicity, antibacterial property, as well as with the functions of promoting tissue growth and wound healing. In addition, the IOF presented interconnected nanopores and high specific surface areas for vascular endothelial growth factor loading, which could further improve its angiogenesis capability. More attractively, as the pH-responsive PAA was incorporated, the IOF patch could report the wound healing status through its real-time structural colors or reflectance spectra.

**Conclusions:**

These features implied the practical value of the multifunctional fish gelatin hydrogel IOFs in clinical wound management.

**Graphical Abstract:**

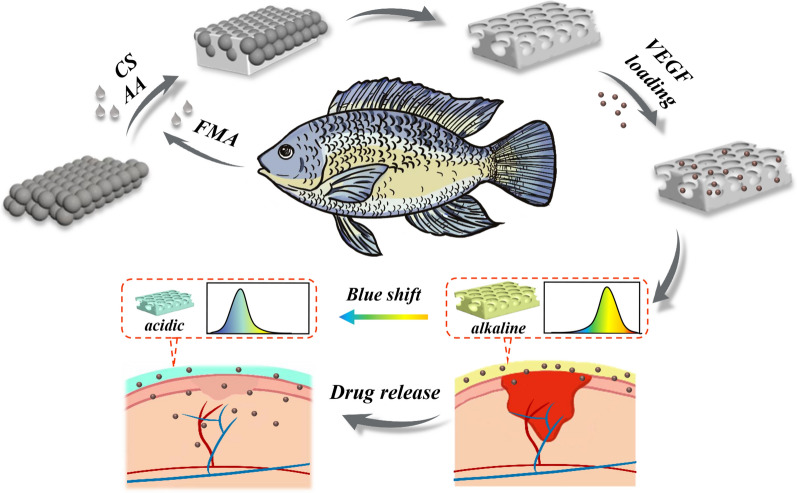

**Supplementary Information:**

The online version contains supplementary material available at 10.1186/s12951-022-01564-w.

## Background

With the increasing attention to the worldwide healthcare hotspot of wound treatment and therapy, a burst number of advanced materials for wound healing have emerged, including electrospun fibers, spongy dressing, nanoparticles, and polymer hydrogels, etc. [1–8]. Among them, gelatin-based hydrogels, fabricated by biopolymers derived from the animal extracellular matrix, are attractive candidates due to their unique capacity of cell attaching [9, 10]. Besides, benefitting from the existence of matrix metalloproteinase responsive peptide motifs, cells are easy to proliferate and spread in the gelatin-based scaffolds [11, 12]. Nowadays, gelatin methacryloyl (GelMA) hydrogel generated by animal skin gelatin has been widely used in many studies of wound healing, with additional functions of drug delivery, anti-infection, hemostasis, biosensing, and so on [13–18]. Although with much progress, most of the gelatin-based materials are derived from bovine or porcine sources, leading to the risks of immune rejection and limited clinical applications [19]. In addition, these animal-derived materials are usually with the simple structure and function of promoting wound healing, lacking effective methods to report wound healing status in real-time. Thus, a multifunctional hydrogel based on a new gelatin source with the function of wound healing and dynamic monitoring is still anticipated.

**Fig. 1:**
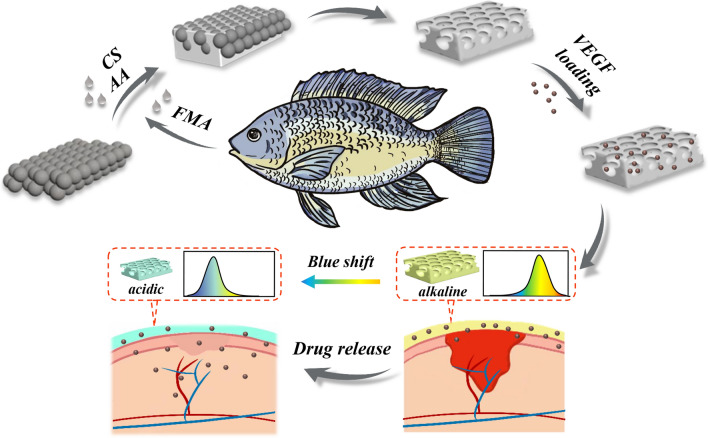
Schematic diagram of the preparation process of the composite IOF and its application in dynamic pH sensing and wound healing

In this work, we propose a novel inverse opal film (IOF) based on a photo-crosslinking fish gelatin hydrogel with the desired wound healing and dynamic monitoring properties, as shown in Fig. 1. The fish gelatin is extracted from the skin of tilapia and is purified by degreasing, impure protein removal, and collagen denaturation [20]. Compared with mammalian gelatin, it possesses prominent superiorities of lower cost, lower immunogenicity and fewer religious restrictions [21]. In contrast, IOFs are flexible materials fabricated by polymers with unique three-dimensional periodic porous structures. They could modulate the propagation of incident light with the visible wavelength range, thus reflecting vivid structural color [22–25]. Especially, when the IOFs were constructed by intelligent responsive polymers, both their internal structure and visual color could respond to external stimuli. This imparts the responsive IOFs with important values in biosensing and health monitoring [26–31]. Therefore, if the responsive composite fish gelatin hydrogel is used to fabricate the IOF, it would generate a new type of material for wound healing with all the anticipated functions.

Herein, we synthesized fish gelatin methacryloyl (FMA) by grafting photocrosslinking groups onto fish gelatin, and constructed pH-responsive IOFs with vibrant structure colors by using the mixture of FMA, chitosan (CS) and polyacrylic acid (PAA) to replicate colloidal crystal templates. Because the molecular structures of fish gelatin are well-retained during the synthetic process, the FMA hydrogel IOFs have brilliant biocompatibility, low immunogenicity, as well as functions of promoting tissue growth and wound healing. Besides, the interconnected nanopores imparted the composite IOFs with a high specific surface area, which could be infiltrated with vascular endothelial growth factor (VEGF), to further obtain antibacterial and angiogenesis capabilities. More importantly, as the PAA was incorporated into the hydrogel system, serving as a pH-responsive unit, the environmental pH value could be detected in real-time by measuring the reflectance spectra of the material. Thus, it is conceivable that the composite IOFs have a further application scenario in the monitoring of wound repairing status. These characters indicate that the multifunctional FMA hydrogel IOFs possess significant prospects for the future development of clinical wound healing.

## Results and discussion

**Fig. 2:**
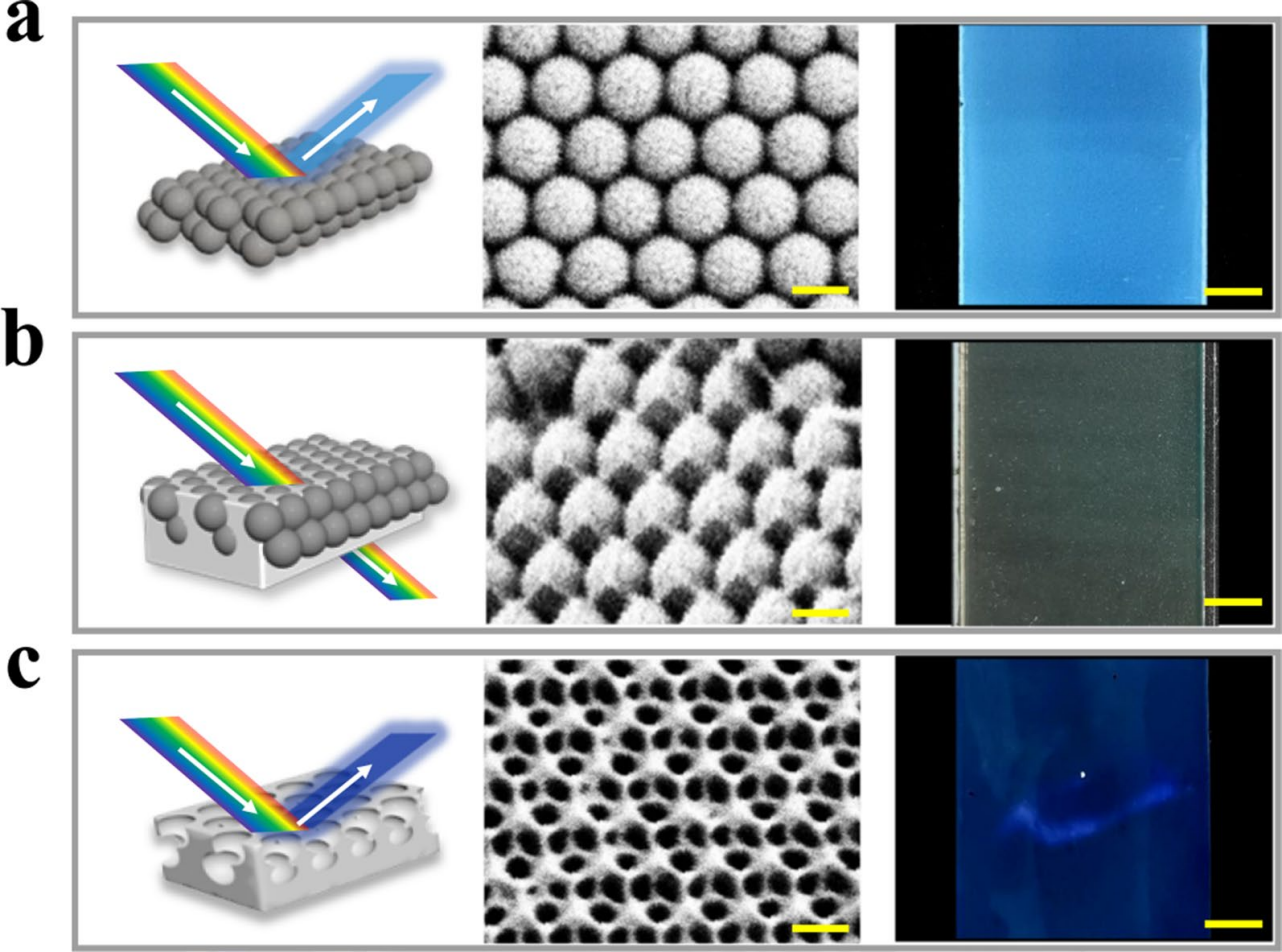
The schematic, SEM image and photograph of **a** the CCA template, **b** the hydrogel penetrated composite material and **c** the resultant IOF. In each panel, the left is the schematic, the middle is the SEM image and the right is the photograph. In each panel, scale bars are 200 nm in the middle and 5 mm in the right

In a typical experiment, FMA and acrylic acid (AA) were first mixed to obtain the pre-gel solution of the composite hydrogel. Colloidal crystal array (CCA) templates were prepared on the clean glass slides. During this procedure, silica nanoparticles with a diameter of 220 nm were self-assembled driven by capillary force, spontaneously formed into a periodically hexagonal packing arrangement (Fig. 2a). After the CCA template was put into a mold as the substrate, the pre-gel solution was then injected in the mold and fully infiltrated the nanoscale voids in CCAs, it was crosslinked to form a hydrogel under ultraviolet (UV) light-triggered polymerization (Fig. 2b). Finally, the silica nanoparticles were etching by hydrofluoric acid, and the hydrogel inverse opal film was obtained (Fig. 2c).

Because of the periodically ordered structure composed of the well assembled silica nanoparticles, the CCA templates on the glass slides showed vivid structural color. Scanning electron microscopy (SEM) was used to confirm its periodically ordered structure of the nanoparticles. The SEM images also suggested that the IOFs successfully replicated the ordered construction and performed interconnected porous structures. These periodic nanopores imparted the resultant films with the property of photonic bandgap (PBG). When incident light illuminated the material surface, the light propagation with a particular wavelength would be modulated and reflected by the nanopores. Typically, obvious structural colors were seen when the wavelength of incident light was in the visible range. The reflection wavelength value (λ) could be calculated approximately by Bragg’s equation:

1 $$\lambda = 1.633{\text{d}}{n_{average}}.$$

In this equation, d refers to the central distance between adjoining nanocrystals or nanopores, while n_average_ represents the material’s average reflectivity. Thus, according to the equation above, when the diameter of nanopores changes, the color of the IOFs would be adjusted.

We prepared a series of IOFs with various structural colors based on the FMA and PAA composited hydrogels and investigated their pH responsiveness. It has been confirmed that the PAA polymer networks could swell/shrink reversibly under the stimulus of pH variation [32, 33]. Under a low pH environment, the dissociation of –COOH groups in the hydrogel was inhibited. The PAA hydrogel shrunk because of the formation of hydrogen bonds between –COOH groups, thus leading to the decrease of volume at this time. As the pH value of the environment increased, –COOH groups gradually dissociated into –COO^−^. The hydrogel would swell in volume because of the resulting increased electrostatic repulsion and osmotic pressure between networks. During these processes, the center distance between nanopores also change, resulting in the change of PBG and visual colors. The rise of reflection wavelength λ showed that increasing pH would lead to a red shift of structural color (Additional file 1: Fig. S1).

Previous researches have reported that the typical pH values are around 4.0–6.0 in the healed wound sites, and are elevated to 7.0 or above for unhealed wounds or infected burns. Thus, we focused on testing the responsiveness of IOFs within the pH range of 4.0–8.0. IOFs with lustrous structural colors of blue, green and red were then obtained by replicating CCA templates with the nanoparticle diameters of 220, 250, and 300 nm, respectively. Subsequently, the color changes of these IOFs under pH stimulation between 4 and 8 were measured. As shown in Additional file 1: Fig. S2, IOFs fabricated by templates with nanoparticle size of 220 nm had the most vivid and wide-range color variation in the mildly acidic and mildly alkaline environments. Thus, CCA templates with a nanoparticle size of 220 nm were selected for further research.

**Fig. 3:**
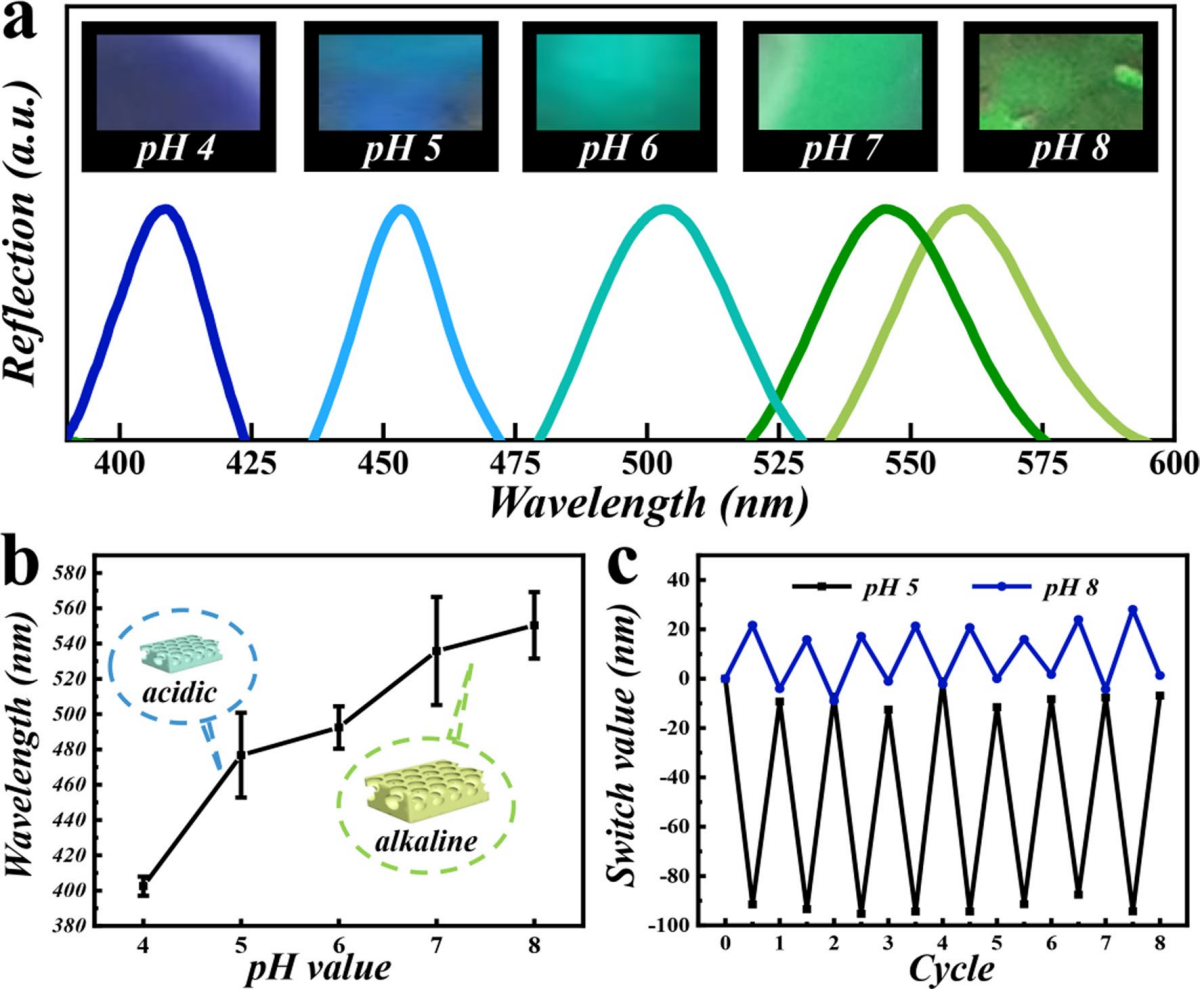
The pH responsiveness of the IOF fabricated by replicating CCA templates with a nanoparticle size of 220 nm. **a** Optical images and variation of the reflection peaks during pH value increasing from 4.0 to 8.0. **b** The changes of reflection peak wavelength upon pH increase. **c** The switch value of the reflection peak of the IOF in the pH 5 and 8 buffers as a function of the pH cycle numbers

The color changes and the variation of reflection peaks of the IOFs fabricated by replicating templates under pH-stimulus were investigated (Fig. 3a). The IOF exhibited blue with a pH of 4.0, attributed to the hydrogel shrinkage caused by the formation of hydrogen bonds between –COOH groups. As the environmental pH progressively raised to 8.0, the IOF swelled, while the color gradually turned green. Meanwhile, the variation of the reflection peaks was detected by using optical spectrometer. During the procedure, with the hydrogel volume increasing, the spacing d between adjacent nanopores increased. As the Bragg’s equation shows, the reflection wavelength λ would also increase (Fig. 3b). More importantly, as shown in Fig. 3c, the hydrogel films still showed stable structural color variation even after several cycles, which could provide reliable results for wound sensing. Therefore, we tested its practical pH sensitive color change in a bacteria-infected wound. The pH values of normal skin and infected wound in animal models were first measured. The pH indicator paper demonstrated that the infected wound showed a significantly alkaline pH value, which resulted in the blue to green structure color change after the IOF was placed on the wound area (Additional file 1: Fig. S3). These results indicated that the intelligent pH-responsive structural color films had potential applications in wound healing monitoring.

**Fig. 4:**
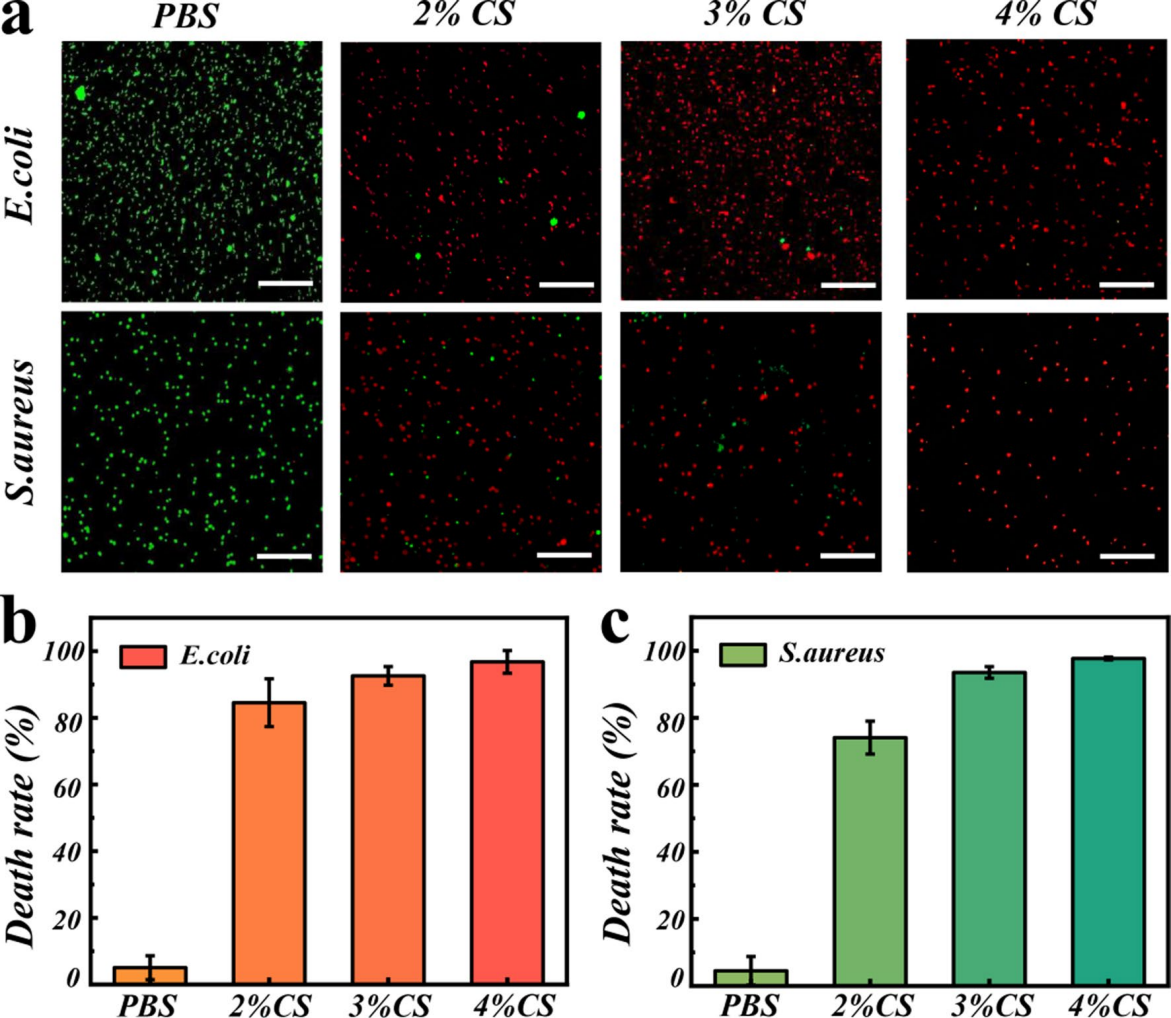
**a** Live/Dead staining of *E. coli* and *S. aureus* treated with PBS, IOF with 2% CS, IOF with 3% CS, and IOF with 4% CS for 24 h. The live and dead bacteria were stained in green and red by SYTO and propidium iodide (PI), respectively. Bacterial death rate of *E. coli* (**b**) and *S. aureus* (**c**) treated with PBS, IOF with 2% CS, IOF with 3% CS, and IOF with 4% CS for 24 h, respectively. Scale bars are 25 μm

Sterilization is important for the repair of infected wounds. CS has been proved to have inherent antibacterial activity attributing to the positively charged amino groups [15]. Thus, we added CS solution into the pre-gel solution and polymerized under UV light. Subsequently, we verified the antibacterial properties of the IOFs loaded with CS by culturing *Escherichia coli* (*E. coli*, a type of Gram-negative bacterium) and *Staphylococcus aureus* (*S. aureus*, a type of Gram-positive bacterium) with the prepared films. The Live/Dead staining results indicated that the bacteria cultured in the PBS buffer almost survived. However, after incubated with the IOF loaded with 2% CS, SYTO staining result revealed that a small number of bacteria survived. Besides, with the increase of CS concentration, the antimicrobial activity increased dramatically. As CS could react with the bacterial cell wall due to the electrostatic adsorption, thereby changing bacterial nutrient intake and leading to bacterial death. Especially when treated with films containing 4% CS, the death rate of the two bacterial almost reached 100% (Fig. 4b, c), which is consistent with the reported result [34]. Similar antibacterial results could be verified by using the standard plate count method. As shown in Additional file 1: Fig. S4, little bacterial colones could be seen in the experimental group compared with the control group. As a consequence, 4% CS was chosen for the following research to impart the films with sufficient antibacterial functionality.

In addition to antibacterial function, the interconnected nanopores have endowed the IOF with an outstanding scaffold for drug infiltrating. For the purpose of verifying the drug loading and releasing abilities of the IOFs loaded with 4% CS, the demo drug with fluorescence mark, fluorescein isothiocyanate-bovine serum albumin (FITC-BSA), was selected. After drug loading, the material was soaked into the phosphate-buffered saline (PBS) buffer. This liquid condition was used to simulate sustained drug release. For the first 12 h, we collected and replaced 100 µL of the buffer solution every 1 h, the time interval for collection would increase during the following 5 days. Microplate reader was used to detect the fluorescence intensity of FITC-BSA in the collected solution, and the amount of the released drug from the IOFs was determined by comparing to the standard curve. It was observed that approximately 30% of the demo drug was finally released in the solution over 144 h, which was less than the reported results, probably due to the different methods of drug loading. These results effectually proved that the IOFs loaded with 4% CS could provide practical and lasting drug treatment for promoting wound repair (Additional file 1: Fig. S5).

Subsequently, we carried in vitro cell experiments to evaluate the biocompatibility of composite film. After incubated with IOFs or the IOFs loaded with CS and VEGF, the cell staining results showed good survivability and normal morphological characteristics of the NIH-3T3 cells (Additional file 1: Fig. S6). Moreover, the hemocompatibility of the obtained films was further assessed by hemolysis tests, as wound dressings must come into contact with blood. In whole blood, IOFs induced nearly no hemolysis in contact with red blood cells (Additional file 1: Fig. S7). These results indicated that IOF was a safe material and suitable for biomedical applications.

**Fig. 5:**
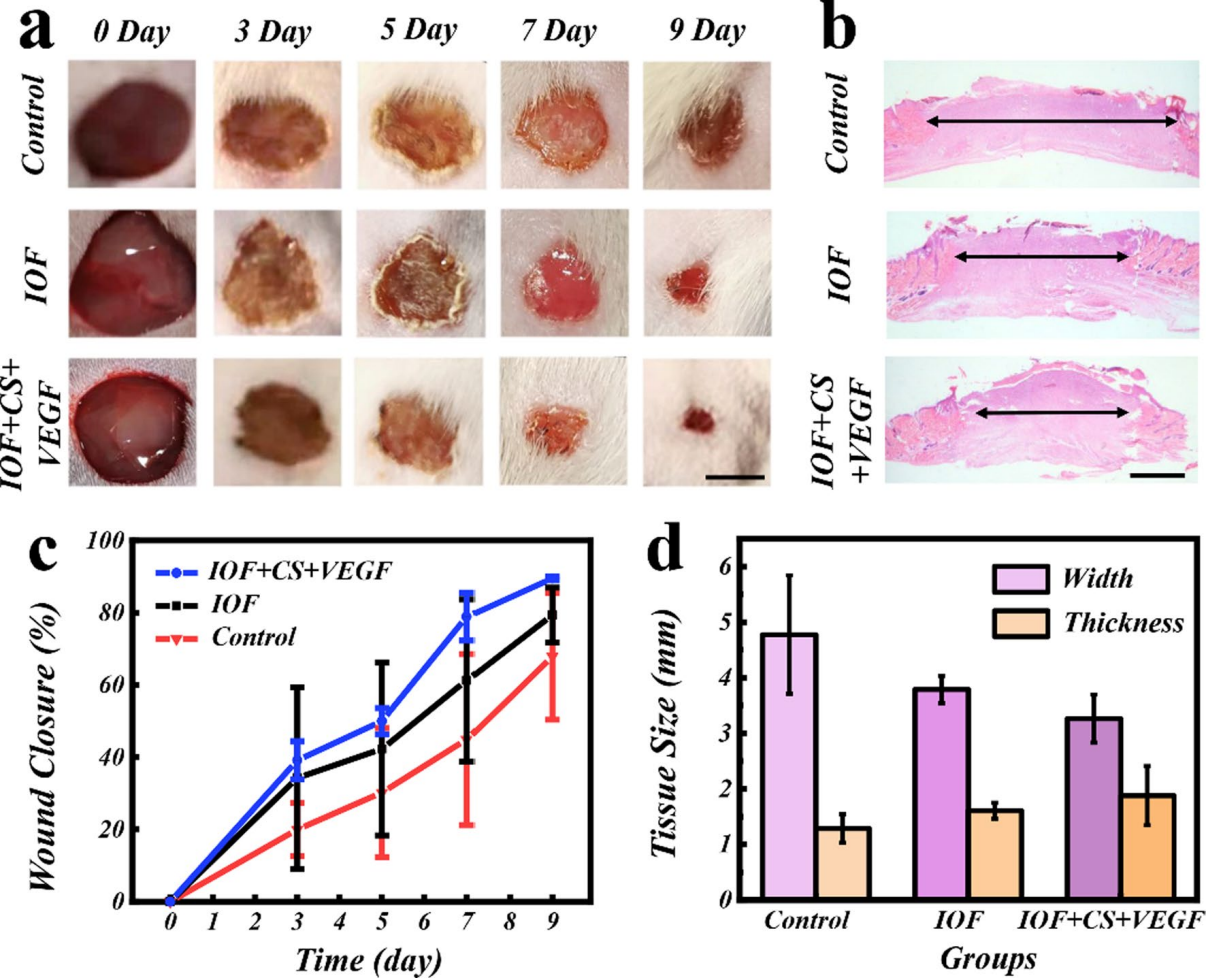
Wound closure process and H&E staining. **a** Representative photographs of the skin wounds treated with PBS solution (control), IOF, the IOF loaded with both CS and VEGF. **b** H&E staining of wounds after 9 days. **c** The statistical graph of the wound closure situation (n = 4). **d** Quantitative analysis of granulation tissue width and thickness (n = 4). Scale bars are 5 mm in **a** and 1 mm in **b**

The practical biomedical value of this multifunctional IOF was further validated in vivo. In a bacterial-infected full-thickness skin defect rat model, the wound with a diameter of 1 cm was established and the bacterial suspension was then injected into the wound area. The Sprague–Dawley (SD) rats were separated into three groups, including the IOF group, the IOF + CS + VEGF group, and PBS buffer group as control. During the wound healing processes, photographs of all the wound sites were recorded by using digital camera on day 0, 3, 5, 7 and 9 for the following detailed analysis (Fig. 5a). In addition, the new granulation tissues were shown by carrying out hematoxylin and eosin (H&E) staining (Fig. 5b). These photos clearly demonstrated that, compared with the control group, the wound in IOF patch-treated groups presented higher closure rate and smaller granulation tissue width. From the quantitative analysis of the wounds closure rate, it could be found that the wound healing efficiency was relatively enhanced benefiting from the function of VEGF (Fig. 5c). Meanwhile, the IOF + CS + VEGF group displayed minimal width of the granulation tissue (3.26 mm), while the PBS treated group demonstrated the maximum width of 4.77 mm. In addition to the contractible wound gap, compared with other groups, the IOF + CS + VEGF group also showed thickest granulation tissue (Fig. 5d). Furthermore, we compared the therapeutic effects of commercial dressings (CD) and the composite IOF. It has been found that the two groups had similar rates of wound closure, while the IOF + CS + VEGF owning a slightly better therapeutic effect (Additional file 1: Fig. S8). From these gross observation and HE staining results, it is believed that the composite film has potential practical application value.

**Fig. 6:**
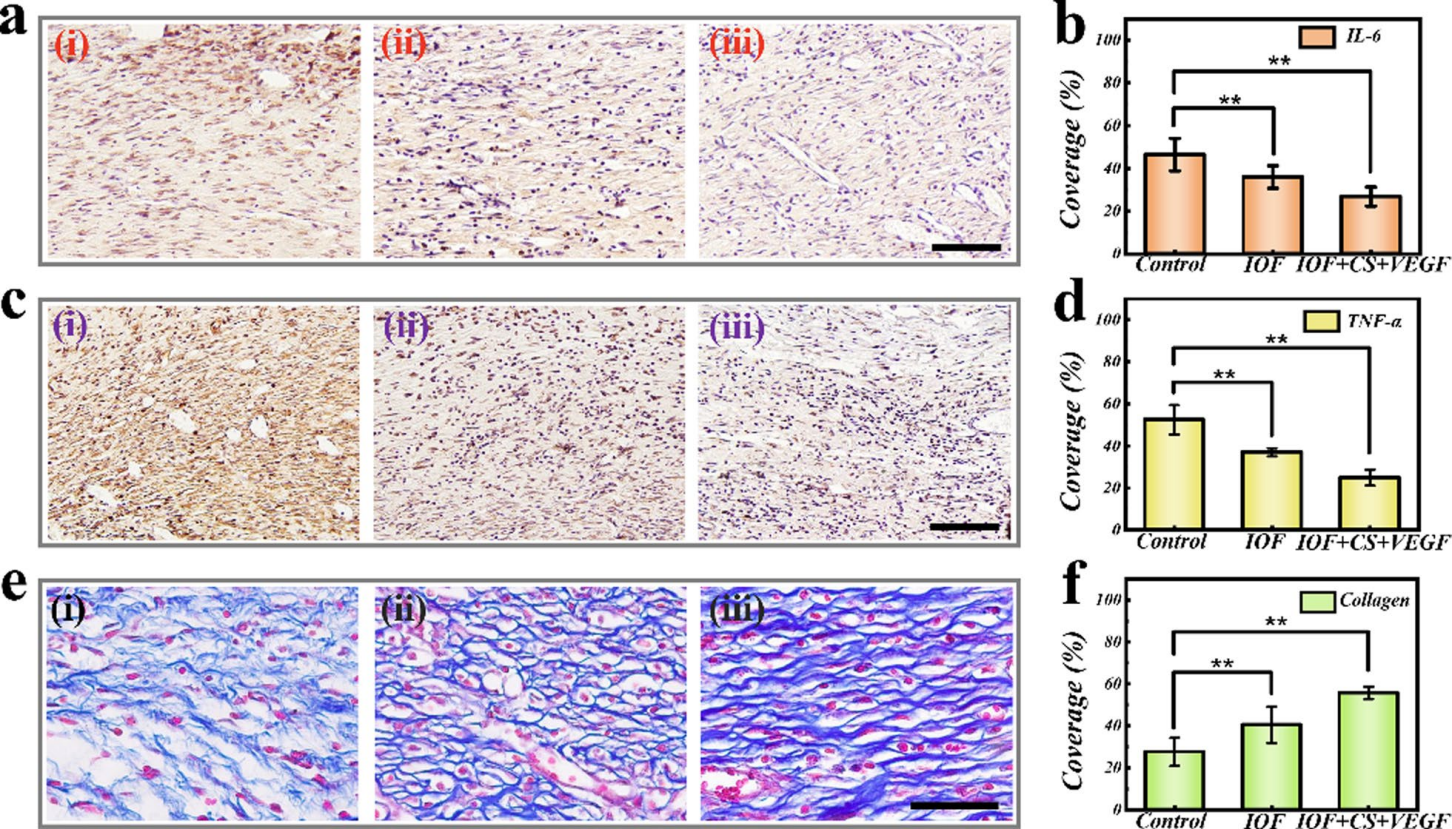
**a** Immunostaining of IL-6 of granulation tissues in different groups. **c** Immunostaining of TNF-α of granulation tissues in different groups. **e** Masson’s trichrome staining for collagen in different groups. Scale bars are all 50 μm. In each panel, (i) is the control group, (ii) is the IOF group, (iii) is the IOF + CS + VEGF group. Statistical analysis of **b** IL-6, **d** TNF-α and **f** collagen deposition in different groups after 9 days of wound repair. *NS* not significant, 0.01 < *p < 0.05, **p < 0.01 (n = 4)

Wounds infected by bacteria may cause serious inflammatory response. At the early stage during wound healing procedure, interleukin-6 (IL-6) and tumor necrosis factor-α (TNF-α) were the two typical inflammatory factors whose expressions were selected as indicators to assess the wound infection level. The expressions were examined by immunohistochemical staining after day 9 (Fig. 6a, c). The stain results showed the highest expression quantity of IL-6 and TNF-α in control group due to the severe inflammatory response. In contrast, little inflammatory factors could be seen in IOF + CS + VEGF group benefiting from the antibacterial activity of CS, which could effectively block bacterial reproduction and protect the wound against bacterial infection. Statistical analysis also demonstrated that the IOF + CS + VEGF group showed the least infection among the three experimental groups (Fig. 6b, d). The in vivo antibacterial properties caused by CS was next assessed 24 h and 48 h after wound appearance by using the standard plate count method. It could be obviously found that compared with the control group, the IOF + CS + VEGF group had less bacterial colony formation, especially after 48 h (Additional file 1: Fig. S9). Additionally, the expression of proliferation cell nuclear antigen (PCNA, a DNA clamp essential for cell replication) and E-cadherin (a type of cell adhesion proteins), were also examined to reveal the cell proliferation and adhesion state, respectively. Additional file 1: Figure S10 presented that the IOF + CS + VEGF group owned the most positive area of PCNA and E-cadherin, indicating the best cell regeneration results. In addition, the IOF + CS + VEGF group showed the best collagen deposition and tissue remodeling result due to the combined treatment of CS and VEGF.

At the last stage during wound healing process, the deposition of collagen in the wound bed is a necessary index that can reflect the tissue remodeling condition. The amount of the collagen deposition was examined by Masson’s trichrome staining (Fig. 6e). The results indicated that, the IOF + CS + VEGF group showed promoted collagen deposition in comparison with the control group, which might be attributed to the sterile environment created by CS. Angiogenesis is also a crucial index to evaluate the remodeling condition of tissue. Thus, we carried out double immunofluorescence staining of CD31 (a typical marker of the vascular endothelial cell) and α-smooth muscle actin (α-SMA, a typical marker of the vascular smooth muscle endothelial cell) to verify new blood vessels formation at the wound site (Additional file 1: Fig. S11). In the control group, a small quantity of new formed blood vessels could be observed, mainly attributed to the high inflammatory level resulted from the bacterial infection. Cell proliferation and differentiation was dramatically impeded. However, the density of new blood vessels at the wound area in the IOF + CS + VEGF group was obviously higher, profiting from the antibacterial property of CS and the stimulating angiogenesis property of VEGF. These features indicated that this synthetic film with multifunctions had great potential applications in the promotion of wound repair.

## Conclusion

In conclusion, we have developed a novel multifunctional IOF patch for wound healing and dynamic monitoring. The patches were composed of fish gelatin methacryloyl, CS, and PAA. It was demonstrated that, due to the existence of pH-responsive PAA, the real-time environmental pH value could be monitored, because the structural color and reflectance spectra of the IOF was red-shifted accompany with the pH increase. Thus, the wound healing status is expected to be monitored using this material in the future. In addition, the well-retained fish gelatin methacryloyl and CS had imparted the film with brilliant biocompatibility and antibacterial capabilities. Moreover, in vivo wound healing studies, owing to the interconnected nanopores and high specific surface area of the IOF patch, VEGF was effectively loaded and sustained released into the wound area, which significantly accelerated the wound healing speed. These results indicated that this multifunctional fish gelatin hydrogel inverse opal film may have a bright future in wound healing.

## Methods

### Materials and animals

Fish gelatin methacryloyl (FMA) and monodispersed silica nanoparticles (220, 250, and 300 nm) were synthesized by Yuanjin Zhao’s group. CS, AA, N,N′-methylenebisacrylamide, 2-hydroxy-2-methylpropiophenone (HMPP), methacrylic anhydride (MA) and hydrofluoric acid were all purchased from Sigma-Aldrich (St. Louis, MO). All the male Sprague–Dawley (SD) rats (180–200 g) of 8 to 12 weeks old were supplied by Comparative Medicine of Jinling Hospital (Nanjing, China). The treatments of the animals were under the recommendations in the Laboratory Animal Care and Use Guidelines. The relevant requests for experiments were reviewed and approved by the Animal Investigation Ethics Committee of Nanjing Drum Tower Hospital, the approval number was 2021AE02013.

### Preparation of FMA

We dissolved fish gelatin (10 g) into PBS at 60 °C, and the gelatin concentration was 10% (w/v). Then, 10 mL MA was added to the fish gelatin solution slowly under stirring. After the solution reacting for 1 h, 400 mL PBS was further added and stirred for 2 h. After dialysis and lyophilization, the FMA was reserved at − 20 °C.

### IOF fabrication

The monodisperse silica nanoparticles were well assembled by using a vertical deposition method and thus CCA templates were fabricated on the glass substrates. Then, for the pre-gel solution, 300 mg FMA, 100 µL AA, 3.3 mg N,N′-methylenebisacrylamide, 20 µL HMPP and 1.6 mL deionized water were added together and mixed extensively. Then the template was soaked by the pre-gel solution and the solution was polymerized under the UV light for 1 min. Afterwards, the IOF was obtained by using hydrofluoric acid to etch and remove CCA template. A SEM (Hitachi S-3000 N) was used to characterize the structure of IOF.

### Test of the pH responsiveness of IOFs

IOFs with structural colors of blue, green and red were fabricated by replicating templates with nanoparticle diameters of 220, 250, and 300 nm, respectively. The prepared IOFs were then stored in deionized water at room temperature. Finally, the IOFs were soaked in phosphate buffer solution with different pH value. Typically, we prepared hydrochloric acid solution and sodium hydroxide solution first, the concentration of both solutions was 0.1 M. Then different proportions of the two solutions were added into 0.1 M potassium hydrogen phosphate buffer solution to prepare different pH value buffer systems from pH 4 to 8. A digital camera (Canon5D Mark III) was used to record the variation of the structural color. Meanwhile, the fiber-optic spectrometer (Ocean Optics; USB2000+) equipped optical microscope (OLYMPUS BX51) was used to detect the reflection spectra of the IOFs soaked in different buffer solutions.

### In vitro antibacterial test

To impart the film with antibacterial capability, CS was first dissolved in a solution of 2% acetic acid to prepare a 4% CS solution. Then, the CS solution was added into the pre-gel solution and polymerized under UV light. We used *E. coli* and *S. aureus* to study the antibacterial capability of the film. The bacteria recovery was conducted in LB liquid medium. After being cultured at 37 °C over night, the bacteria were collected after centrifugation and resuspended in sterile PBS to a turbidity of 0.5. The IOF loaded with 4% CS was first cut into 1 cm in diameter pieces. Two pieces were put into the hole of the 48-well plate and 500 µL of *E. coli* and *S. aureus* suspension was added, respectively. The bacteria were treated by the material for 24 h at 37 °C. SYTO and PI was then added into the suspension for the Live/Dead stain. After stained with SYTO and PI for 30 min and 2 min, respectively, a fluorescence microscope was used to observe the survival of bacterial.

### Drug release experiments

The IOF loaded with 4% CS was dried over 1 h at room temperature and the dehydrated film was then soaked in the 1 mg/mL of FITC-BSA solution for 12 h. After that, the film was transferred into 1 mL of PBS solution at 37 °C. For the first 12 h, we collected and replaced 100 µL of the PBS buffer every 1 h, the time interval for collection could increase during the following experiment. The collected buffer solution was added to a 96-well plate and a microplate reader was used to record the fluorescence intensity of FITC-BSA. Meanwhile, we established a standard curve of the fluorescence intensity and the drug concentration.

### Biocompatibility test

15% fetal bovine serum (Gibco, USA) and 1% penicillin–streptomycin double antibiotics were added into DMEM/high glucose medium (Gibco, USA). The NIH-3T3 fibroblast cells were cultivated in the medium under the condition of 5% CO_2_ and 37 °C. The films were immersed in sterile PBS and sterilized under the UV light over night. The films were then cleaned three times with sterile PBS and put on the bottom of a 48-well plate. The cell suspension was then added to the plate and co-cultured with IOF and IOF + CS + VEGF. After that, the MTT assay was performed after 0, 1 and 3 d of cell culture, and the cell activity was reflected by the OD value. Meanwhile, the films were also co-cultured with 3T3 cells. After being cultured for 3 d and stained by Calcein-AM, we used a fluorescence microscope to observe the number and morphologies of cells. The blood compatibility was also evaluated by using hemolysis assay.

$${\text{Hemolysis}}\;{\text{ratio}}\;\left( \% \right) = 100\% \times \left( {{\text{Ab}}{{\text{s}}_{{\text{sample}}}} - {\text{Ab}}{{\text{s}}_{{\text{negative}}}}} \right)/\left( {{\text{Ab}}{{\text{s}}_{{\text{positive}}}} - {\text{Ab}}{{\text{s}}_{{\text{negative}}}}} \right)\left( {{\text{Abs}}\;{\text{was}}\;{\text{the}}\;{\text{absorbance}}\;{\text{at}}\;540\;{\text{nm}}} \right).$$

### Wound healing study in vivo

An infected full-thickness skin defect model was first built to verify the function of multifunctional IOFs in wound repair. After all rats were anesthetized, their back hairs were shaved and back skin with 1 cm diameter was removed. Then 100 µL of bacterial suspension was injected to the wound site. The animals were separated into three groups and treated with PBS, IOF, VEGF and CS-loaded IOF, respectively, four parallel samples were set up in each group. The wounds condition was recorded on day 0, 3, 5, 7 and 9. And we collected the regrowing tissues after days 9.

### Histology, immunohistochemistry, and immunofluorescence staining

After the regenerated tissues were collected, the tissues were immersed in 4% paraformaldehyde solution for 48 h for fixation. After immersed into 50% ethanol solution for 1 h, 70% ethanol solution for 4 h, 80% ethanol solution for 3 h, 95% ethanol solution for 3 h, the obtained dehydrated tissues were embedded by paraffin. Then the samples were sliced into slices with a thickness of 5 µm for the following study. H&E staining was used to demonstrate the thickness of the granulation tissues. The immunohistochemical staining was carried out to evaluate the inflammation level. The collagen deposition was demonstrated by Masson’s trichrome staining. Finally, double tissue immunofluorescence staining of CD31 and α-SMA was utilized to show new blood vessels formation condition.

### Statistical analysis

For each data, at least three independent experiments were performed, then the mean value and standard deviations were calculated and SPSS software was used for statistical analyses. The significance was analyzed with one-way ANOVA with significance as follows: NS not significant, 0.01 < *p < 0.05, **p < 0.01. As p < 0.05 was considered to have significant differences.

## Supplementary Information

**Additional file 1: Figure S1.** (a) pH-driven deprotonation equilibria of PAA polymers. (b) The change of the volume and structure color of the IOF under pH-stimulus. (c) The red shift of the reflection peak during the pH increasing process. **Figure S2.** Optical images of the pH-responsive structural color change of the IOFs fabricated by templates with different nanoparticle sizes. **Figure S3.** The photographs of pH indicator paper and IOF put onto (a) normal skin and (b) infected wounds of SD rats. (c) the reference for pH values in the range of 1–14. The scale bars are 5 mm. **Figure S4.** Photographs of *E. coli* and *S. aureus* colonies treated with PBS, and IOF loaded with 4% CS. Scale bars are 2 cm. **Figure S5.** (a) The standard curve of FITC-BSA in the PBS buffer solution, R^2^ > 0.999. (b) Cumulative release curve of FITC-BSA in IOF loaded with 4% CS. **Figure S6.** Live staining of NIH-3T3 cells on (a) glass dish, (b) IOF and (c) IOF loaded with 4% CS and VEGF. (d) The statistical graph of cell viability. Scale bars are 100 μm. **Figure S7.** Hemolysis tests of IOF and IOF + CS + VEGF. **Figure S8.** (a) Representative photographs of the wound closure process treated with commercial dressing (CD), and IOF + CS + VEGF. (b) The statistical graph of the wound closure situation (n = 4). **Figure S9.** In vivo antibacterial test of (a) control group and IOF + CS + VEGF group. Scale bars are 2 cm. **Figure S10.** (a) Immunostaining of PCNA of granulation tissues in different groups. (c) Immunostaining of E-cadherin of granulation tissues in different groups. Scare bars are 50 μm. **Figure S11.** Double immunofluorescence staining of neovascularization, CD31(+) structures (red) were surrounded by α‐smooth muscle actin positive cells (green) in different groups: (a) Control, (b) IOF, (c) IOF + CS + VEGF. Scale bars are 50 μm. (d) Statistical analysis of vessel density in different groups after 9 days of wound repair. NS not significant, 0.01 < *p < 0.05, **p < 0.01 (n = 4). **Figure S12.** Photographs of the skin wounds treated with (a) PBS solution (control), (b) IOF, (c) the IOF loaded with both CS and VEGF. Scale bar is 5 mm.

## Data Availability

All data generated or analyzed during this study are included in this manuscript and its additional file.
