# Supplementary material for: Multifunctional fish gelatin hydrogel inverse opal films for wound healing
Source: J Nanobiotechnology. 2022 Aug 2;20:355. doi: 10.1186/s12951-022-01564-w (PMC9344764; doi:10.1186/s12951-022-01564-w)
Supplement: Supplementary file 1 — Additional file 1: Figure S1. (a) pH-driven deprotonation equilibria of PAA polymers. (b) The change of the volume and structure color of the IOF under pH-stimulus. (c) The red shift of the reflection peak during the pH increasing process. Figure S2. Optical images of the pH-responsive structural color change of the IOFs fabricated by templates with different nanoparticle sizes. Figure S3. The photographs of pH indicator paper and IOF put onto (a) normal skin and (b) infected wounds of SD rats. (c) the reference for pH values in the range of 1–14. The scale bars are 5 mm. Figure S4. Photographs of E. coli and S. aureus colonies treated with PBS, and IOF loaded with 4% CS. Scale bars are 2 cm. Figure S5. (a) The standard curve of FITC-BSA in the PBS buffer solution, R2 > 0.999. (b) Cumulative release curve of FITC-BSA in IOF loaded with 4% CS. Figure S6. Live staining of NIH-3T3 cells on (a) glass dish, (b) IOF and (c) IOF loaded with 4% CS and VEGF. (d) The statistical graph of cell viability. Scale bars are 100 μm. Figure S7. Hemolysis tests of IOF and IOF + CS + VEGF. Figure S8. (a) Representative photographs of the wound closure process treated with commercial dressing (CD), and IOF + CS + VEGF. (b) The statistical graph of the wound closure situation (n = 4). Figure S9. In vivo antibacterial test of (a) control group and IOF + CS + VEGF group. Scale bars are 2 cm. Figure S10. (a) Immunostaining of PCNA of granulation tissues in different groups. (c) Immunostaining of E-cadherin of granulation tissues in different groups. Scare bars are 50 μm. Figure S11. Double immunofluorescence staining of neovascularization, CD31(+) structures (red) were surrounded by α‐smooth muscle actin positive cells (green) in different groups: (a) Control, (b) IOF, (c) IOF + CS + VEGF. Scale bars are 50 μm. (d) Statistical analysis of vessel density in different groups after 9 days of wound repair. NS not significant, 0.01 < *p < 0.05, **p < 0.01 (n = 4). Figure S12. Photographs of the [file 12951_2022_1564_MOESM1_ESM.docx]

Additional file 1 for

**Multifunctional fish gelatin hydrogel inverse opal films for wound healing**

*Xinyue Cao^1^, Zhuohao Zhang^1^, Lingyu Sun^1^, Zhiqiang Luo^1^, and Yuanjin Zhao^1,2,^**

^1^ Department of Rheumatology and Immunology, Nanjing Drum Tower Hospital, School of Biological Science and Medical Engineering, Southeast University, Nanjing 210096, China

^2^ Oujiang Laboratory (Zhejiang Lab for Regenerative Medicine, Vision and Brain Health); Wenzhou Institute, University of Chinese Academy of Sciences, Wenzhou, Zhejiang 325001, China

Corresponding author：Yuanjin Zhao

**Fig. S1.** (a) pH-driven deprotonation equilibria of PAA polymers. (b) The change of the volume and structural color of the IOF under pH-stimulus. (c) The redshift of the reflection peak during the pH increasing process.

**Fig. S2**. Optical images of the pH-responsive structural color change of the IOFs fabricated by templates with different nanoparticle sizes.

**Fig. S3**. The photographs of pH indicator paper and IOF put onto (a) normal skin and (b) infected wounds of SD rats. (c) The reference for pH values in the range of 1-14. The scale bars are 5 mm.

**Fig. S4**. Photographs of *E. coli* and *S. aureus* colonies treated with PBS, and IOF loaded with 4% CS. Scale bars are 2 cm.

**Fig. S5.** (a) The standard curve of FITC-BSA in the PBS buffer solution, R^2^＞0.999. (b) Cumulative release curve of FITC-BSA in IOF loaded with 4% CS.

**Fig. S6.** Live staining of NIH-3T3 cells on (a) plastic dish, (b) IOF and (c) IOF loaded with 4% CS and VEGF. (d) The statistical graph of cell viability. Scale bars are 100 μm.

**Fig. S7.** Hemolysis tests of IOF and IOF+CS+VEGF.

**Fig. S8.** (a) Representative photographs of the wound closure process treated with commercial dressing (CD), and IOF+CS+VEGF. (b) The statistical graph of the wound closure situation (n=4).

**Fig. S9.** *In vivo* antibacterial test of (a) control group and (b) IOF+CS+VEGF group. Scale bars are 2 cm.

**Fig. S10.** (a) Immunostaining of PCNA of granulation tissues in different groups. (b) Immunostaining of E-cadherin of granulation tissues in different groups. Scare bars are 50 μm.

**Fig. S11.** Double immunofluorescence staining of neovascularization, CD31(+) structures (red) were surrounded by α‐smooth muscle actin positive cells (green) in different groups: (a) Control, (b) IOF, (c) IOF+CS+VEGF. Scale bars are 50 μm. (d) Statistical analysis of vessel density in different groups after 9 days of wound repair. NS not significant, 0.01 < *p < 0.05, ** p < 0.01 (n=4).

**Fig. S12.** Photographs of the skin wounds treated with (a) PBS solution (control), (b) IOF, (c) the IOF loaded with both CS and VEGF. Scale bar is 5 mm.
